# *Aloe vera* attenuated liver injury in mice with acetaminophen-induced hepatitis

**DOI:** 10.1186/1472-6882-14-229

**Published:** 2014-07-08

**Authors:** Duangporn Werawatganon, Sittikorn Linlawan, Kessarin Thanapirom, Kanjana Somanawat, Naruemon Klaikeaw, Rungsun Rerknimitr, Prasong Siriviriyakul

**Affiliations:** 1 Departments of Physiology, Faculty of Medicine, Chulalongkorn University, Bangkok 10330, Thailand; 2 GI Unit, Department of Internal Medicine, Faculty of Medicine, Chulalongkorn University, Bangkok 10330, Thailand; 3 Department of Pathology, Faculty of Medicine, Chulalongkorn University, Bangkok 10330, Thailand

**Keywords:** *Aloe vera*, Liver injury, Serum transaminases, MDA, GSH, Interleukin

## Abstract

**Background:**

An overdose of the acetaminophen causes liver injury. This study aims to examine the anti-oxidative, anti-inflammatory effects of *Aloe vera* in mice with acetaminophen induced hepatitis.

**Methods:**

Male mice were randomly divided into three groups (n = 8 each). Control group were given orally distilled water (DW). APAP group were given orally N-acetyl-P-aminophenol (APAP) 400 mg/kg suspended in DW. *Aloe vera*-treated group were given orally APAP and *Aloe vera* (150 mg/kg) suspended in DW. Twenty-four hours later, the liver was removed to determine hepatic malondialdehyde (MDA), hepatic glutathione (GSH), the number of interleukin (IL)-12 and IL-18 positive stained cells (%) by immunohistochemistry method, and histopathological examination. Then, the serum was collected to determine transaminase (ALT).

**Results:**

In APAP group, ALT, hepatic MDA and the number of IL-12 and IL-18 positive stained cells were significantly increased when compared to control group (1210.50 ± 533.86 *vs* 85.28 ± 28.27 U/L, 3.60 ± 1.50 *vs* 1.38 ± 0.15 nmol/mg protein, 12.18 ± 1.10 *vs* 1.84 ± 1.29%, and 13.26 ± 0.90 *vs* 2.54 ± 1.29%, *P* = 0.000, respectively), whereas hepatic GSH was significantly decreased when compared to control group (5.98 ± 0.30 *vs* 11.65 ± 0.43 nmol/mg protein, *P* = 0.000). The mean level of ALT, hepatic MDA, the number of IL-12 and IL-18 positive stained cells, and hepatic GSH in *Aloe vera*-treated group were improved as compared with APAP group (606.38 ± 495.45 *vs* 1210.50 ± 533.86 U/L, *P* = 0.024; 1.49 ± 0.64 *vs* 3.60 ± 1.50 nmol/mg protein, *P* = 0.001; 5.56 ± 1.25 *vs* 12.18 ± 1.10%, *P* = 0.000; 6.23 ± 0.94 *vs* 13.26 ± 0.90%, *P* = 0.000; and 10.02 ± 0.20 *vs* 5.98 ± 0.30 nmol/mg protein, *P* = 0.000, respectively). Moreover, in the APAP group, the liver showed extensive hemorrhagic hepatic necrosis at all zones while in *Aloe vera*-treated group, the liver architecture was improved histopathology.

**Conclusions:**

APAP overdose can cause liver injury. Our result indicate that *Aloe vera* attenuate APAP-induced hepatitis through the improvement of liver histopathology by decreased oxidative stress, reduced liver injury, and restored hepatic GSH.

## Background

An overdose of the analgesic drug acetaminophen causes liver injury in experimental animals and humans. The toxicity has been shown to be initiated by cytochrome P450 metabolism to N-acetyl-p-benzoquinone imine (NAPQI) [1,2]. The high reactivity of NAPQI with sulfhydryl groups results in depleting glutathione in hepatocytes, followed by covalent binding to intracellular proteins [3,4]. Although it has been shown that the relative amount of covalent binding is correlated with the development of the toxicity [3], it is also suggested that the covalent binding is not sufficient for the toxicity [5-7]. Extensive studies were thus focused on covalent binding to specific proteins as a trigger of the toxicity. A number of proteins have been identified as targets of NAPQI by the immunological techniques [8] and recent proteomics [9]. Because development of various mitochondria dysfunctions has been observed with acetaminophen toxicity, which include inhibition of respiration, a decrease in hepatic ATP levels, a decrease in membrane potential, a loss of mitochondrial Ca^2+^[10-13], it was proposed that mitochondria was primary target of the reactive metabolite. Indeed, some of the target proteins were localized in mitochondria fraction, including glutamate dehydrogenase, aldehyde dehydrogenase, carbamyl phosphate synthetase-I and ATP synthetase α-subunit [8,9]. These enzyme activities in the mitochondrial fraction were decreased partially [14,15], probably as consequence of covalent binding, while it is also estimated that only the loss of the any single enzyme activity could not explain mitochondria-mediated acetaminophen hepatotoxicity, it is thus presumed that the toxicity is accounted for by combination of covalent binding to several functional proteins and/or by secondary event following the covalent binding.

A previous study demonstrated that APAP overdose administration showed significantly increase in serum transaminase, hepatic malondialdehyde (MDA), and decreased hepatic glutathione (GSH). Histological examination showed a severe centrilobular hepatic necrosis with fatty changes [16-18].

Somanawat *et al.*[18] recently reported that administration of APAP to mice resulted in a significant increase in serum IL-12 and IL-18 expression and significant decrease in curcumin treatment, by using enzyme-linked immunosorbent assay (ELISA) method.

Preliminary data obtained in animal models of APAP-induced liver injury showed that the treatment with curcumin could be effective in limiting liver injury, but the exact mechanism of these effects is still largely undefined.

Interleukin-12 (IL-12) was originally indentified as a natural killer (NK) cells stimulatory factor, a disulfide–linked heterodimeric cytokine composed of 35 and 40 Ku subunits. IL-12 secreted principally by antigen presenting cells (APC), such as macrophages, B cells, and dendritic cells, activates NK cells and T cells to produce interferon-γ (IFN-γ), and augments their cytotoxic activity and proliferation [19].

Interleukin-18 (IL-18), originally known as interferon-γ (IFN-γ)-inducing factor (IGIF), is a cytokine that shares structural and functional properties with interleukin-1 (IL-1) [20,21]. This cytokine is mainly produced by activated macrophages, but may also be expressed by Kupffer cells, T cells, B cells, keratonocytes, astrocytes, and osteoblasts [22]. Like IL-1, IL-18 is synthesized as an inactive precursor (pro-IL-18, 24 kDa), which is cleaved by interleukin-1 β-converting enzyme (ICE or caspase-1) into an active 18 kDa mature form [22-24].

*Aloe vera* (*Aloe barbadensis Mill*) is classified in family of *Aloaceae*, originated in the dry areas of Africa, Asia, and Southern Europe, especially in the Mediterranean regions. *Aloe vera* and other species of *Aloe* are succulent and xerophytic plants that are adapted to living in areas with little water. These plants possess extensive water storage tissue in their leaves, the part of the plant which is used for its therapeutic properties [25]. The largest component (60%) in the dry matter, are the carbohydrates (soluble sugar and complex polysaccharides).

Historically, *Aloe vera* has been known as a traditional folklore medicine for the treatment of many diseases and sicknesses. *Aloe vera* is a well-known anti-inflammatory and wound-healer, accelerating the rapid growth of epithelial tissue. Davis *et al.*[26] examined evidence that *Aloe vera* is effective in treating wounds and reducing inflammation by the action of mannose-6-phosphate (a major sugar in Aloe gel), which they found to be an active growth substance. *Aloe vera* is known to have a marked effect in the treatment of scar tissue and the prevention of scar formation following injury to the skin. This is because *Aloe vera* stimulates cell production through the activity of the amino acids, which are the basis for new cell formation, and also, due to the ability of its enzymes, promotes regeneration at the deepest layers of the skin. *Aloe vera* has also been used in the treatment of burns. It is suggested that lectin may be responsible for the therapeutic effect of the gel on burns [27]. *Aloe vera* can be used successfully in the general treatment of skin ulcers, including mouth ulcers, cold sores (herpes simplex), and leg ulcers. This is possibly due to the anti-virucidal effect of the *Aloe vera* gel at concentrations of about 80% [28]. Blitz *et al.*[29] reported the use of *Aloe vera* gel internally taken to treat peptic ulcers and disturbances of the gastrointestinal tract. They attributed the gel effect to coacervation of pepsin inhibition of hydrochloric acid secretion and a general detoxifying effect. In the field of dentistry, *Aloe vera* has been used to treat a variety of dental conditions and has been found to relieve pain and accelerate healing after periodontal flap surgery [30]. The use of *Aloe vera* gel has also been described in veterinary medicines. The gel extract has been used in the treatment of a number of external conditions in many animals [31,32]. These conditions include ringworm, allergies, abscesses, fungal infections, various types of inflammation, pain, and itching. Anti-oxidative and immunosuppressive activities of *Aloe vera* have been indicated [33,34].

There are currently limited studies investigating the effects of *Aloe vera* on APAP-induced liver injury. However, the effect of *Aloe vera* on IL-12 and IL18, in mice with APAP-induced hepatitis has never been investigated. Therefore, in this study, we determined whether *Aloe vera* could attenuate liver injury in mice with APAP-induced hepatitis and also influence IL-12 and IL-18, which is a proinflammatory cytokines.

## Methods

### Animal care and diets

All experiments and procedures carried out on the animals were approved by the Ethics Committee of Faculty of Medicine, Chulalongkorn University, Bangkok, Thailand. Male ICR strain mice, weighing 25–30 grams, were purchased from the National Laboratory Animal Center, Mahidol University (Bangkok, Thailand). Mice were housed individually in stainless steel metabolic cages and maintained in an environmentally controlled room (25 ± 1°C) under standard condition (12-hours dark–light cycle). Each cage was equipped with a feeder and drinking water to allow animal free access to food and drinking water. Mice were fasted for 18 hours starting from 4.00 p.m. with access to drinking water *ad libitum* before the experiment.

### N-acetyl-P-aminophenol (APAP) and *Aloe vera* preparations

A single dose of APAP tablet (Tylenol®) was suspended in distilled water (DW) that was freshly prepared before gavaging at the concentration of 400 mg/kg body weight (b.w.). Lyophilized powder of *Aloe vera* was suspended in DW at the concentration of 150 mg/kg b.w. (Lipo Chemical Co, United States). The doses of APAP and *Aloe vera* were used according to Somanawat *et al.*[18] and minor adapted from Prabjone *et al.*[35], respectively. Oral median lethal dose (LD_50_) values of APAP in multiple animal models are around 400 to 900 mg/kg b.w. (mice) and more than 2,000 mg/kg b.w. (rat, rabbit, and guinea pig) [36]. Oral LD_50_ value of *Aloe vera* in mice was estimated to be 4.8 g/kg b.w. [37].

### Preparation of fresh *Aloe vera* powder from *Aloe vera* gel

Leaves of 1-year old growing *Aloe vera* were cut and washed thoroughly with water to exclude the drained juice containing aloin. The edges of the leaves were cut, and then split to extract the pulp which contains active mucopolysaccharide substance. The pulp was then mixed in a blender and sieved through fine gauze. *Aloe vera* gel was kept in a refrigerator. The gel was changed into powder form by freeze drying using a lyophilizer. The *Aloe vera* powder was reconstituted into gel form and dispended in DW before use.

### Experimental design

After acclimatization for 7 days, mice were randomly divided into three experimental groups: control (n = 8), N-acetyl-P-aminophenol or APAP (n = 8), and *Aloe vera-*treated group (n = 8) as follows.

Control group: Mice were given DW at the volume of 0.5 ml/mice orally *via* an intragastric tube once a day on the 1^st^ day and served as normal control.

APAP group: Mice were given APAP at the concentration of 400 mg/kg b.w. suspended in DW at the volume of 0.5 mL/mice orally *via* an intragastric tube once a day on the 1^st^ day.

*Aloe vera-*treated group: Mice were given APAP at the concentration of 400 mg/kg b.w. and *Aloe vera* at the concentration of 150 mg/kg b.w. suspended in DW at the volume of 0.5 mL/mice orally *via* an intragastric tube once a day on the 1^st^ day.

### Mice anesthesia and care

Pre-anesthetic care reduces the incidence of complications that can occur in the course of anesthesia by ensuring the choice of the most suitable technique and regimen. As a rule, animals purchased from external sources should be singly housed for 1 or 2 weeks both to acclimate the animals and to allow time before an experiment for animal care personnel to observe and evaluate the health of the animals. Personnel attending the mice must be trained to handle the animals gently but firmly because this handling has a strong influence on the animals’ physiological functions. Pre-anesthetic fasting in mice is generally deemed unnecessary because the animals cannot vomit. Conversely, prolonged fasting can cause hypoglycemia due to their low hepatic glycogen reserve. Some concern about anesthetizing mice that have a full stomach is linked to limited diaphragmatic excursion and to gastric blood pooling during digestion.

Anesthesia in laboratory animals is a state of unconsciousness, analgesia, muscle relaxation, and a-reflexia. Induction of general anesthesia in mice can be achieved by a variety of drugs and techniques. The most commonly used anesthetics in mice include the injectable agent pentobarbital. In mice, injectable anesthetics can best be administered via intraperitoneal (i.p.) route. The injection volume should be carefully considered according to the available route: adequate volumes by the i.p. route range from 0.1 to 1 ml. During sedation and anesthesia, it is imperative to carefully monitor and support mice body temperature, heart and respiratory rates, mucous membranes, and the degree of CNS depression. Anesthesia is considered adequate when the animal stays still quietly, is unresponsive to external stimuli, and has constant heart and respiratory rates. In mice the absence of the palpebral reflex suggests a fair anesthetic depth.

On the 2^nd^ day or twenty-four hours after the experiment, laparotomy was performed in mice under anesthesia with i.p. injection of thiopental (50 mg/kg b.w.). The abdominal wall was incised and liver was removed and wash with cold normal saline (4°C- 8°C). The liver was chopped into small pieces, frozen in liquid nitrogen, and stored at -80°C to examine hepatic lipid peroxidation (MDA) and GSH. The hepatic MDA was quantified by thiobarbituric acid reaction as described by Ohkawa *et al.*[38]. The hepatic GSH was quantified by GSH Assay Kit (Cayman Chemical Company, United States). The remaining liver was isolated, fixed in 10% formalin solution to examine histopathology and inflammatory cytokines, interleukin (IL)-12 and IL-18, by immunohistochemistry method.

Then whole blood was withdrawn from heart. The blood was allowed to coagulate at room temperature for 2 hours and centrifuged for 20 minutes at 1000 × g to obtain serum. The serum was collected to examine transaminase, serum glutamic oxaloacetic transaminase (SGOT) and serum glutamic pyruvic transaminase (SGPT) by Reflotron®Plus Clinical Chemistry analyzer (Roche, Switzerland).

### Hepatic lipid peroxidation (MDA) assay

Lipid peroxidation of liver in mice using thiobarbituric acid (TBA) was measured by a modified method of Ohkawa *et al.*[38]. One gram of liver tissue was homogenized in 3 ml of 50 mM potassium phosphate buffer (pH 7.0). To 0.3 ml of liver homogenated in test tube, 1.5 ml of 10% trichloroacetic acid (TCA) solution and 1.5 ml of 0.8% TBA solution were added. The mixture was boiled in waterbath at 95°C for 60 minutes and then cooling with water at room temperature. After centrifugation at 1000 × g for 15 minutes, the absorbance of sample was measured at 532 nm. 1, 1, 3, 3 tetramethoxy propane (TMP) was used as a standard of MDA. The MDA content was calculated in comparison with a standard MDA curve and was expressed as nmol/mg protein.

### Hepatic anti-oxidant (GSH) assay

Cayman’ GSH assay utilizes an optimized enzymatic recycling method, using GSH reductase, for the quantification of GSH. The sulfhydryl group of GSH reacts with 5, 5′-ditrio-*bis*-(2-nitrobenzoic acid) (DTNB), or Ellman’s reagent and produces a yellow colored 5-thio-2-nitrobenzoic acid (TNB). The mixed disulfide, GSTNB (between GSH and TNB) that is concomitantly produced, is reduced by GSH reductase to recycle the GSH and produce more TNB. The production of TNB is directly proportional to this recycling reaction which is in turn directly proportional to the concentration of GSH in the sample. The optical density (O.D.) of TNB is then measured at 405–414 nm using a microplate reader, which provides an accurate estimation of GSH in the sample.

### Histologic measurements of liver pathology

Sample of the liver were excised and transferred to formalin and later processed by routine techniques prior to embedding in paraffin. Sections were cut at the thickness of 5 μm and stained with hematoxylin and eosin (H and E). An experienced pathologist blinded to the experiment evaluated all samples. All histopathological changes were observed under light microscope. Hepatic necroinflammation score in each section was graded according to the criteria described by Brunt *et al.*[39] from 0 to 3 as follow; Score 0 = No hepatocyte injury/inflammation; Score 1 = Sparse or mild focal zone 3 hepatocyte injury/inflammation; Score 2 = Noticeable zone 3 hepatocyte injury/inflammation; Score 3 = Severe zone 3 hepatocyte injury/inflammation.

### Immunohistochemistry for inflammatory cytokines (IL-12 and IL-18) measurement

The liver sections were deparaffinized with xylene and ethanol for ten minutes. After water washing, sections retrieved the antigen with citrate buffer pH 6.0 in microwave for thirteen minutes. Next, 3% H_2_O_2_ and 3% normal horse serum were performed on the slides to block endogenous peroxidase activity for five minutes and blocked nonspecific binding for twenty minutes, respectively. Then, the primary antibody used for IL-12 and IL-18, monoclonal antibodies against the Mouse IL-12 and IL-18, were then applied at a dilution of 1:100 for IL-12 (R and D Systems, Inc., United States) and 1:200 for IL-18 (Gene Tex, United States) respectively for one hour at room temperature and incubated with the secondary antibody for IL-12 (1:100; Dako, Denmark) and IL-18 (ready-to-use; Dako, Denmark) for thirty minutes. When the development of the color with diaminobenzidine (DAB) was detected, the slides were counterstained with hematoxylin.

Under light microscopy (Nikon E50i, Nikon Corporation, Japan), the number of positive cells presented dark brown stained nuclei of liver Kupffer cells. Images were obtained at × 20 and × 40 magnification field from each sample. Ten images from two sections per animal were analyzed. The numbers of positive stained cells were counted manually using Point tool in the IMAGE-PRO® PLUS software program (version 6.1). A five hundred of macrophages or liver Kupffer cells were counted for each sample. The results were expressed as the number of IL-12 and IL-18 Kupffer positive stained cells (%) calculating from this equation:

The number of positive stained cells (%) $=\left(\frac{\mathrm{number}\;\mathrm{of}\;\mathrm{nuclei}\;\mathrm{stained}\;\mathrm{cells}}{\mathrm{number}\;\mathrm{of}\;\mathrm{examined}\;\mathrm{cells}}\right)\times 100\%$.

### Statistical analysis

All data are presented as mean ± SD. We evaluated group differences with one-way analysis of variance (one-way ANOVA) followed by Tukey PostHoc comparision by using SPSS for windows version 17.0 (SPSS Inc., Chicago, IL, United States). A *P*-value of < 0.05 was considered significant.

## Results

### Change in serum SGOT and SGPT levels

**Figure 1:**
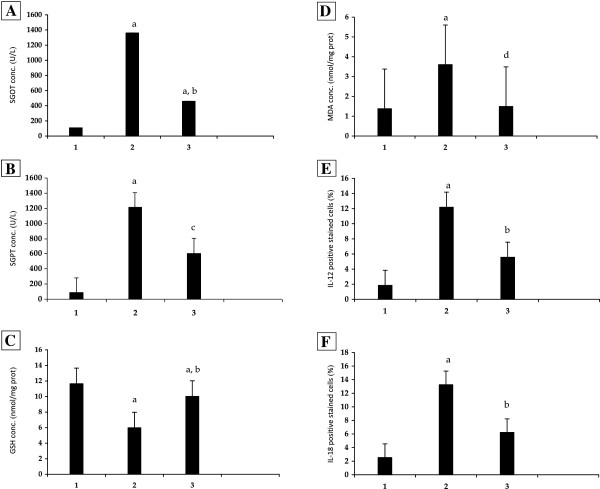
**The effects of *Aloe vera *on biological parameters in mice with N-acetyl-P-aminophenol (APAP) overdose. A**: Serum glutamic oxaloacetic transaminase (SGOT). **B**: Serum glutamic pyruvic transaminase (SGPT). **C**: Hepatic glutathione (GSH). **D**: Hepatic malondialdehyde (MDA). **E**: Interleukin (IL)-12. **F**: Interleukin (IL)-18. ^a^*P* = 0.000 *vs* control group; ^b^*P* = 0.000, ^c^*P* = 0.024, and ^d^*P* = 0.001 *vs* N-acetyl-P-aminophenol (APAP) group. 1: Control; 2: APAP; 3: *Aloe vera-*treated.

Serum transaminase, SGOT and SGPT, are often used as markers as their increase indicates liver damage. The serum SGOT and SGPT levels were significantly increased in APAP group when compared with control group (1361.20 ± 290.26 *vs* 107.80 ± 38.66 U/L and 1210.50 ± 533.86 *vs* 85.28 ± 28.27 U/L, *P* = 0.000) and significantly decreased in *Aloe vera*-treated group compared with APAP group (458.62 ± 228.21 *vs* 1361.20 ± 290.26 U/L, *P* = 0.000 and 606.38 ± 495.45 *vs* 1210.50 ± 533.86 U/L, *P* = 0.024) (Table 1 and Figure 1A, B).

**Table 1:** Summary of parameters in all experimental groups

**Group/Parameters**	***n***	**IL-12 (%)**	**IL-18 (%)**	**SGOT**	**SGPT**	**MDA**	**GSH**
Control	8	1.84 ± 1.29	2.54 ± 1.29	107.80 ± 38.66	85.28 ± 28.27	1.38 ± 0.15	11.65 ± 0.43
APAP	8	12.18 ± 1.10^a^	13.26 ± 0.90^a^	1361.20 ± 290.26^a^	1210.50 ± 533.86^a^	3.60 ± 1.50^a^	5.98 ± 0.30^a^
*Aloe vera*-treated	8	5.56 ± 1.25^b^	6.23 ± 0.94^b^	458.62 ± 228.21^a,b^	606.38 ± 495.45^c^	1.49 ± 0.64^d^	10.02 ± 0.20^a,b^

The data were presented as the mean ± SD of serum SGOT, serum SGPT, hepatic GHS, hepatic MDA, and the number of IL-12 and IL-18 liver Kupffer positive stained cells (%) in all experimental groups (n = 8). ^a^*P* = 0.000 *vs* control group; ^b^*P* = 0.000, ^c^*P* = 0.024, and ^d^*P* = 0.001 *vs* N-acetyl-P-aminophenol (APAP) group.

### Change in hepatic GSH level

GSH is essential for detoxification of APAP metabolites and NAPQI that cause hepatic necroinflammation. Hepatic GSH was significantly decreased in APAP group when compared with control group (5.98 ± 0.30 *vs* 11.65 ± 0.43 nmol/mg protein, *P* = 0.000). These were significantly restored in *Aloe vera*-treated group when compared with APAP group (10.02 ± 0.20 *vs* 5.98 ± 0.30 nmol/mg protein, *P* = 0.000) (Table 1 and Figure 1C).

### Change in hepatic MDA level

Hepatic MDA, which is the main metabolite in the intracellular lipid peroxidation reaction significantly increased in APAP group when compared with control group (3.60 ± 1.50 *vs* 1.38 ± 0.15 nmol/mg protein, *P* = 0.000). These were significantly decreased in *Aloe vera-*treated group when compared with APAP group (1.49 ± 0.64 *vs* 3.60 ± 1.50 nmol/mg protein, *P* = 0.001) (Table 1 and Figure 1D).

### Change in IL-12 and IL-18 levels

The expressions of both IL-12 and IL-18 in macrophages or liver Kupffer cells were determined by immunohistochemistry (IHC) method and showed as dark brown stained nuclei of this cell.

**Figure 2:**
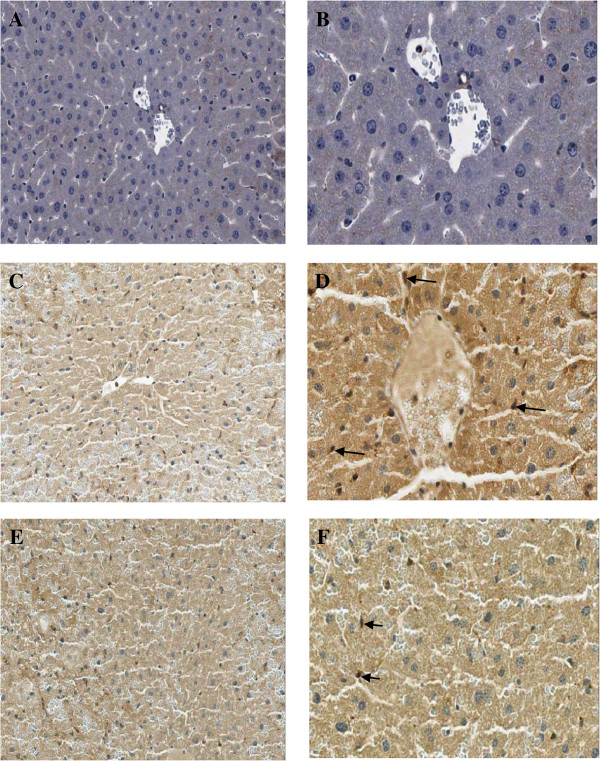
**Immunohistochemical staining of IL-12 antibody in the representative tissue specimens. A-B**: Control. **C-D**: APAP. **E-F**: *Aloe vera*-treated. Images were obtained at × 20 (A, C, and E) and × 40 (B, D, and F). DAB staining was used to highlight liver Kupffer cells in each section (dark brown stain, arrows).

**Figure 3:**
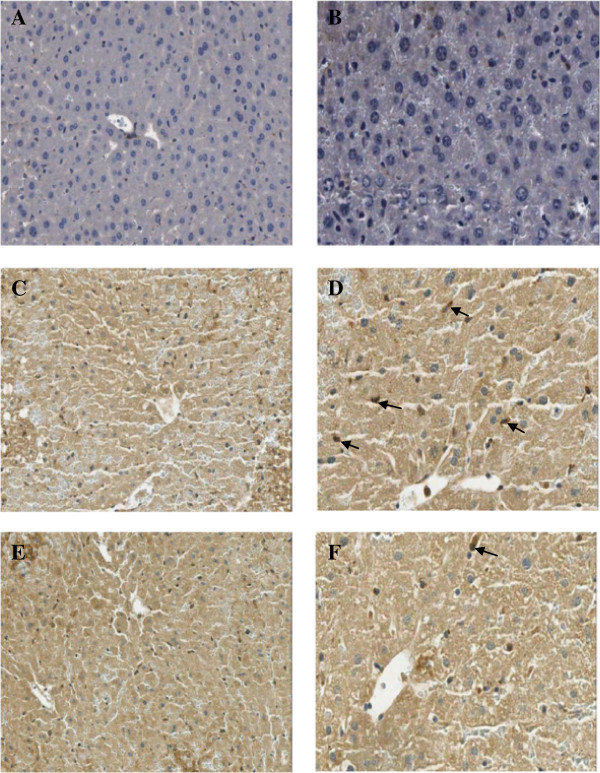
**Immunohistochemical staining of IL-18 antibody in the representative tissue specimens. A-B**: Control. **C-D**: APAP. **E-F**: *Aloe vera*-treated. Images were obtained at × 20 **(A, C, and E)** and × 40 **(B, D, and F)** DAB staining was used to highlight liver Kupffer cells in each section (dark brown stain, arrows).

The data of both IL-12 and IL-18 expressions in all groups were given in Table 1 and Figures 1E, F, 2, 3. From the result, the number of IL-12 and IL-18 positive stained cells in the liver Kupffer cells of APAP group was significantly higher than control group (12.18 ± 1.10 *vs* 1.84 ± 1.29% and 13.26 ± 0.90 *vs* 2.54 ± 1.29%, *P* = 0.000, respectively). In contrast, *Aloe vera*-treated group (150 mg/kg BW) decreased the number of positive stained cells significantly when compared with APAP group (5.56 ± 1.25 *vs* 12.18 ± 1.10% and 6.23 ± 0.94 *vs* 13.26 ± 0.90%, *P* = 0.000, respectively).

### Histopathology

**Figure 4:**
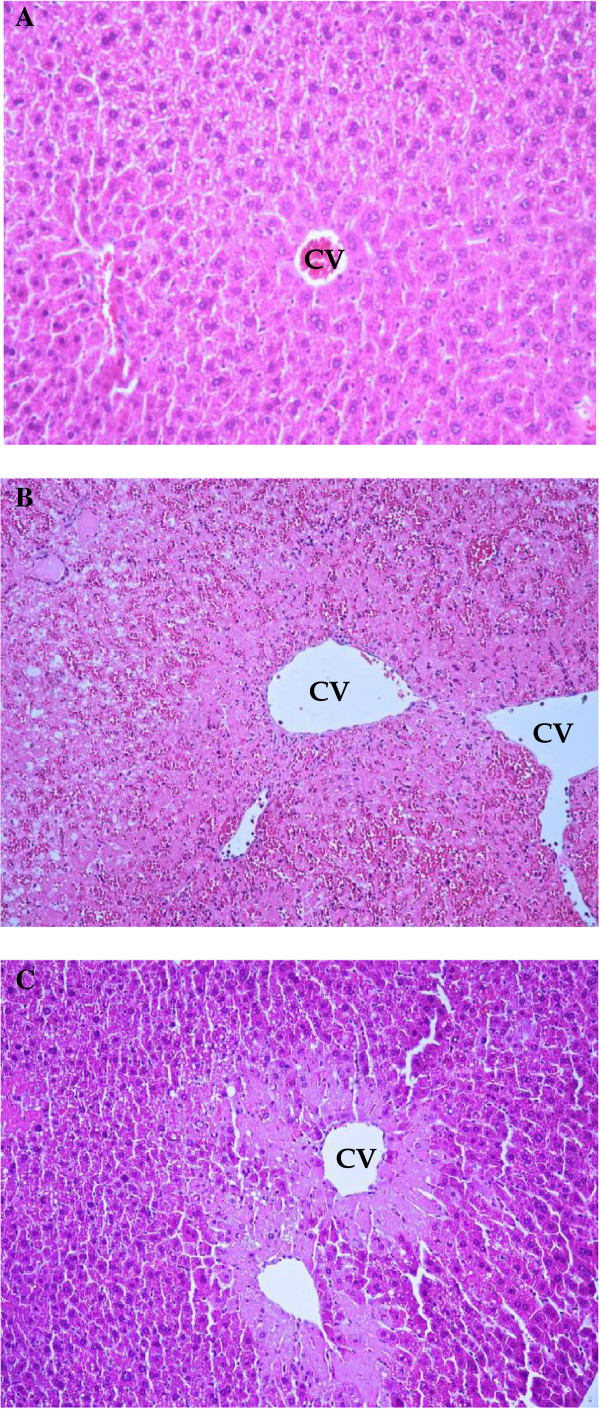
**The effects of *Aloe vera *improved liver histopathology (hematoxylin and eosin stain). A**: Control group showed normal hepatic architecture (× 40). **B**: N-acetyl-P-aminophenol (APAP) group showed acute centrilobular hemorrhagic hepatic necrosis through all zones (zones 1, 2, and 3) (× 40). **C**: *Aloe vera*-treated group showed focal necrosis with mild fatty changes and hepatic architecture was well preserved with limited hepatic change (× 40). Arrows indicate hepatic necrosis and CV indicate central vein.

In control group, hepatic necroinflammation score was 0 (n = 8) showed normal histology. In APAP group (n = 8), the hepatic necroinflammation score was 2 (n = 2) and 3 (n = 6) that showed moderate and severe injury, respectively. In *Aloe vera*-treated group (n = 8), the hepatic necroinflammation score of was 1 (n = 4), 2 (n = 3), and 3 (n = 1) that showed improvement of severity (Table 2). Histopathological examination of APAP group when compared with control group showed acute centrilobular hemorrhagic hepatic necrosis involving all zones. The improvement of liver pathology revealed in *Aloe vera*-treated group when compared with APAP group that showed focal necrosis and the classical hepatic architecture was preserved (Figure 4).

**Table 2:** Summary of the hepatic necroinflammation score in all experimental groups

**Group**	**n**	**Hepatic necroinflammation scores**			
		**0**	**1**	**2**	**3**
Control	8	8	0	0	0
APAP	8	0	0	2	6
*Aloe vera*-treated	8	0	4	3	1

Data are presented as the number of mice exhibiting each score of hepatic necroinflammation (n = 8). Hepatic necroinflammation score in each section was graded according to the criteria described by Brunt *et al.*[39] from 0 to 3 as follow; Score 0: No hepatocyte injury/inflammation; Score 1: Sparse or mild focal zone 3 hepatocyte injury/ inflammation; Score 2: Noticeable zone 3 hepatocyte injury/inflammation; Score 3: Severe zone 3 hepatocyte injury/inflammation. APAP: N-acetyl-P-aminophenol.

## Discussion

APAP-induced liver injury has reported in several mechanisms [40-42]. APAP undergoes metabolism to glucuronide and sulfate conjugates, which are excreted in the urine. Normally, only a small proportion (about 5%) of APAP is oxidized to NAPQI that is detoxified by hepatic GSH. Excessive doses of APAP (>7.5 g/day in adult or > 150 mg/kg in children) lead to the saturation of glucuronic acid and/or sulfate pathways shunting more APAP into the cytochrome P450 (CYP) system [43]. The hepatorenal toxicity occurs when GSH reserves are depleted. NAPQI binds cellular proteins including oxidation of thiol groups in mitochondria leading to mitochondrial permeability transition. The ensuing mitochondrial dysfunction generates profound oxidative stress and also facilitates peroxynitrate formation. These events culminate in nuclear and cytoplasmic swelling, cytoplasmic vacuolization, and mitochondrial swelling of both hepatocytes and sinusoidal endothelial cells that finally cause apoptosis [44]. Immune mechanisms have been increasingly implicated in APAP-induced hepatotoxicity [45]. APAP-induced hepatocyte death leads to the release of free DNA and the subsequent activation of Toll-like receptor, increase production of interleukin-1 (IL-1) and other cytokines leading to neutrophil recruitment. Lipid peroxidation of cell membrane results in decreasing of membrane fluidity, inability to restore ionic gradients that finally cause cellular swelling or tissue inflammation.

This study demonstrated that administration of N-acetyl-P-aminophenol **(**APAP**)** or paracetamol overdose induced hepatitis results in oxidative stress, liver injury, liver histopathology, and hepatic GSH depletion in mice. GSH is essential for detoxification of APAP metabolites and NAPQI that cause hepatic necroinflammation. These could be attenuated by treatment with *Aloe vera* extraction. Our result corresponds to previous observations studied in mice and rats models [16-18].

*Aloe vera* contains polysaccharide active substances “Acemannan” and other anti-oxidative agents that prior animal-model studies reporting about hepatoprotective effect in alcoholic hepatitis or chemical-induced hepatotoxicity such as carbon tetrachloride, lindane, azoxymethan, etc. [46]. In this study, we found that *Aloe vera* extract results in minimization of hepatic MDA level, which is the main metabolite in the intracellular lipid peroxidation reaction. The ability of *Aloe vera* to exert a significant and prevention on the oxidative damage caused by APAP overdose when given in mice confirms its anti-oxidative damage effect.

It has been reported that IL-12 is produced by dendritic cells, monocytes, Langerhans cells, neutrophils, and keratinocytes [19]. IL-18 is produced by activated macrophages, keratinocytes, intestinal epithelial cells, oeteoblasts, adrenal cortex cells, and the murine diencephalon. IL-18 is synthesized as a precursor 24 kilodalton molecule without a signal peptide and must be cleaved to produce an active molecule. IL-1 converting enzyme (or caspase-1) cleaves peo-IL-18, producing the mature bioactive peptide that is readily released from cells [22,23,47-49].

Importantly, both of these cytokines are produced from Kupffer cells or macrophages in the liver. It may be possible that inflammation is due to hepatocytes releasing cytokines into the blood stream. Furthermore, IL-12, in combination with IL-18, causes inflammation *via* the activity of interferon-gamma (IFN-γ), which is produced by T-lymphocyte and NK cells.

Tangkijvanich *et al.*[48] reported that serum IL-12 level was significantly higher in patients with hepatocellular carcinoma (HCC) than in healthy controls by using enzyme-linked immunosorbent assay (ELISA) method. Furthermore, they found that its levels were correlation with IL-18 level, suggesting that these cytokines may act synergistically in the anti-tumor activity.

Our previous study demonstrated that curcumin administration caused significantly elevated inflammatory mediators, IL-12 and IL-18 levels, in the serum of mice with APAP induced-hepatitis by using ELISA method [18]. However, the effect of *Aloe vera* on inflammatory mediators, IL-12 and IL-18, in animal models with APAP-induced hepatitis has never been investigated.

In this study, we found significant rises in inflammatory mediators, IL-12 and IL-18, in the liver Kupffer cells of the APAP group compared to the control group through increases the number of IL-12 and IL-18 positive stained cells (%) by using immunohistochemistry method.

Thus, the detailed mechanisms of both IL-12 and IL-18 involving in liver Kupffer cells inflammation should be further investigated. Moreover, treatment with *Aloe vera* could attenuate liver injury in mice with APAP-induced hepatitis. Despite its inconclusive mechanism of action, we clearly demonstrated that *Aloe vera* has a protective and beneficial effect on APAP-induced hepatitis in mice. Further study requires for confirm our hypothesis and benefit of *Aloe vera* on the expression of these inflammatory mediators in APAP-induced hepatitis in animal model.

## Conclusions

APAP could induce hepatitis through increases in oxidative stress, hepatic GSH depletion, liver injury, and liver histopathology. *Aloe vera*, an anti-inflammatory, anti-oxidant, and curing wound herbal substance, could prevent these adverse events and might be used as an adjunctive therapy for APAP-induced hepatitis.

## Competing interests

The authors declare that they have no competing interests.

## Authors’ contributions

DW carried out the design of the study, performed the statistical analysis, and edited the manuscript. SL carried out the liver injury study and participated in the design of the study. KT carried out the oxidative stress (MDA), anti-oxidative stress (GSH) studies, and participated in the design of the study. KS carried out the immunohistochemistry study, participated in the design of the study, and wrote the manuscript. NK participated in the pathological examination. RR participated in the design of the study. PS participated in the design of the study and edited the manuscript. All authors read and approved the final manuscript.

## Pre-publication history

The pre-publication history for this paper can be accessed here:

http://www.biomedcentral.com/1472-6882/14/229/prepub
